# Chronic Air Pollution Exposure during Pregnancy and Maternal and Fetal C-Reactive Protein Levels: The Generation R Study

**DOI:** 10.1289/ehp.1104345

**Published:** 2012-02-03

**Authors:** Edith H. van den Hooven, Yvonne de Kluizenaar, Frank H. Pierik, Albert Hofman, Sjoerd W. van Ratingen, Peter Y.J. Zandveld, Jan Lindemans, Henk Russcher, Eric A.P. Steegers, Henk M.E. Miedema, Vincent W.V. Jaddoe

**Affiliations:** 1 Generation R Study Group, Erasmus Medical Center, Rotterdam, the Netherlands; 2 Urban Environment and Safety, Netherlands Organisation for Applied Scientific Research (TNO), Utrecht, the Netherlands; 3 Department of Epidemiology,; 4 Department of Clinical Chemistry,; 5 Department of Obstetrics and Gynaecology, and; 6 Department of Paediatrics, Erasmus Medical Center, Rotterdam, the Netherlands

**Keywords:** air pollution, C-reactive protein, dispersion modeling, inflammation, nitrogen dioxide, particulate matter, pregnancy

## Abstract

Background: Exposure to air pollution has been associated with higher C-reactive protein (CRP) levels, suggesting an inflammatory response. Not much is known about this association in pregnancy.

Objectives: We investigated the associations of air pollution exposure during pregnancy with maternal and fetal CRP levels in a population-based cohort study in the Netherlands.

Methods: Particulate matter (PM) with an aerodynamic diameter ≤ 10 μm (PM_10_) and nitrogen dioxide (NO_2_) levels were estimated at the home address using dispersion modeling for different averaging periods preceding the blood sampling (1 week, 2 weeks, 4 weeks, and total pregnancy). High-sensitivity CRP levels were measured in maternal blood samples in early pregnancy (*n* = 5,067) and in fetal cord blood samples at birth (*n* = 4,450).

Results: Compared with the lowest quartile, higher PM_10_ exposure levels for the prior 1 and 2 weeks were associated with elevated maternal CRP levels (> 8 mg/L) in the first trimester [fourth PM_10_ quartile for the prior week: odds ratio (OR), 1.32; 95% confidence interval (CI): 1.08, 1.61; third PM_10_ quartile for the prior 2 weeks: OR, 1.28; 95% CI: 1.06, 1.56]; however, no clear dose–response relationships were observed. PM_10_ and NO_2_ exposure levels for 1, 2, and 4 weeks preceding delivery were not consistently associated with fetal CRP levels at delivery. Higher long-term PM_10_ and NO_2_ exposure levels (total pregnancy) were associated with elevated fetal CRP levels (> 1 mg/L) at delivery (fourth quartile PM_10_: OR, 2.18; 95% CI: 1.08, 4.38; fourth quartile NO_2_: OR, 3.42; 95% CI: 1.36, 8.58; *p*-values for trend < 0.05).

Conclusions: Our results suggest that exposure to air pollution during pregnancy may lead to maternal and fetal inflammatory responses.

C-reactive protein (CRP) is an acute-phase reactant and a frequently used marker of low-grade systemic inflammation whose levels increase in response to both infectious and noninfectious stimuli (Gabay and Kushner 1999). CRP levels have been suggested to increase during pregnancy because of the maternal inflammatory response to the pregnancy (Thornton 2010; von Versen-Hoeynck et al. 2009). Among pregnant women, elevated CRP levels have been associated with adverse outcomes such as preterm delivery, preeclampsia, and fetal growth restriction (Ernst et al. 2011; Guven et al. 2009; Lohsoonthorn et al. 2007; Pitiphat et al. 2005; Tjoa et al. 2003). Additionally, elevated CRP levels in umbilical cord blood have been reported in infants being born small for gestational age (Amarilyo et al. 2011; Trevisanuto et al. 2007).

CRP levels might increase in response to air pollution exposure. Previous studies have linked air pollution exposure to increased CRP levels in various populations, including healthy adults, diseased subjects, and elderly subjects, but results have been inconsistent (Brauner et al. 2008; Chuang et al. 2007; Delfino et al. 2008; Diez Roux et al. 2006; Dubowsky et al. 2006; Forbes et al. 2009; Riediker et al. 2004; Rudez et al. 2009; Seaton et al. 1999; Steinvil et al. 2008; Zuurbier et al. 2011). Only one study investigated the associations of air pollution exposure with CRP levels in pregnant women (Lee et al. 2011). Associations of maternal air pollution exposure with fetal CRP levels have not yet been examined. This is of interest because induction of systemic inflammation has been proposed as one potential biological mechanism through which air pollution could result in adverse pregnancy outcomes (Kannan et al. 2006; Slama et al. 2008).

Therefore, we investigated the associations of maternal exposure to particulate matter (PM) with an aerodynamic diameter ≤ 10 μm (PM_10_) and nitrogen dioxide (NO_2_) during pregnancy with maternal and fetal CRP levels in a population-based cohort study among 6,508 mother–child pairs living in an urban area in the Netherlands.

## Methods

*Design.* This study was embedded in the Generation R Study, a population-based prospective cohort study from early pregnancy onward in the city of Rotterdam, the Netherlands, which has been described previously in detail (Jaddoe et al. 2010). Mothers enrolled between 2001 and 2005. The study protocol was approved by the Medical Ethical Committee of Erasmus Medical Center, Rotterdam. Written informed consent was obtained from all mothers.

Of the 8,880 prenatally enrolled women, air pollution exposure estimates were available for 7,899 mothers (89%). For 981 mothers, air pollution exposure data could not be assessed because of incomplete address history or because they had moved outside the study area before delivery (Jaddoe et al. 2010). Mothers with a twin pregnancy (*n* = 85), abortion (*n* = 7), or intrauterine death (*n* = 12) were excluded. Of the mothers with live singleton births and their infants, a CRP measurement in maternal blood and/or cord blood was available for 6,508 mother–infant pairs. Median gestational age at enrollment was 13.1 weeks (range, 5.1–38.4 weeks). We excluded mothers and infants with extremely high CRP values (> 100 mg/L, *n* = 4, and > 20 mg/L, *n* = 8, respectively), because these concentrations are likely to reflect acute inflammatory processes due to specific infectious causes. Associations between air pollution exposure and CRP levels were analyzed in 5,067 mothers with a maternal CRP measurement in the first trimester and in 4,450 infants with a fetal CRP measurement at delivery [for a flow chart, see Supplemental Material, Figure S1 (http://dx.doi.org/10.1289/ehp.1104345)].

*Air pollution exposure.* Individual exposures to PM_10_ and NO_2_ during pregnancy were assessed at the home address, using a combination of continuous monitoring data and geographic information system–based dispersion modeling techniques, taking into account both the spatial and temporal variation in air pollution. The method has been previously described in detail (van den Hooven et al. 2011, 2012). In brief, annual average concentrations of PM_10_ and NO_2_ for the years 2001–2006 were assessed for all addresses in the study area, using the three Dutch national standard methods for air quality modeling (Netherlands Ministry of Infrastructure and the Environment 2007). Hourly concentrations of PM_10_ and NO_2_ were derived, taking into account hourly wind conditions and fixed temporal patterns in the contribution of air pollution sources. Subsequently, the hourly concentrations were adjusted for background concentrations, using hourly air pollution measurements from three continuous monitoring stations. We obtained full residential history of the participants, which showed that approximately 13% of the women moved at least once during pregnancy. Based on participants’ home addresses, we derived average exposure estimates for different periods preceding the day of blood sampling (in first trimester or at delivery): 1 week (days 1–7), 2 weeks (days 1–14), and 4 weeks (days 1–28). Additionally, we estimated average exposure for the total pregnancy period (conception until delivery).

*High-sensitivity CRP levels.* Maternal venous blood samples were collected in early pregnancy (median, 13.2 weeks of gestation; range, 4.5–17.9 weeks). Sampling of venous umbilical cord blood was carried out by midwives and obstetricians immediately after delivery (median, 40.1 weeks of gestation; range, 27.6–43.6 weeks). Blood samples were transported to the regional laboratory for processing and storage at –80°C (Jaddoe et al. 2007). High-sensitivity CRP (hs-CRP) concentrations were measured in EDTA plasma samples at the Department of Clinical Chemistry of the Erasmus Medical Center in 2009. We measured hs-CRP because traditional clinically used CRP methods lack the sensitivity in low ranges needed for predicting future risk of events in apparently healthy individuals (Roberts et al. 2001). We analyzed hs-CRP levels using an immunoturbidimetric assay on the Architect System (Abbot Diagnostics BV, Hoofddorp, the Netherlands). The total precision (interassay variation) for hs-CRP was 0.9% at 12.9 mg/L and 1.3% at 39.9 mg/L. The lowest level of detection was 0.2 mg/L. Elevated maternal CRP concentrations were defined as > 8 mg/L (~ 83rd percentile), a cutoff point that has been associated with adverse pregnancy outcomes in previous studies (Catov et al. 2007; Pitiphat et al. 2005). Elevated fetal CRP levels were defined as > 1 mg/L (~ 97th percentile), a threshold that has been associated with neonatal infection (Kordek et al. 2008).

*Covariates.* Medical records were used to obtain information on date of birth, gestational age at birth, fetal sex, and birth weight. Information on maternal age, educational level, ethnicity, parity, and first-trimester infectious or inflammatory disease (doctor-consulted) was obtained by a questionnaire at enrollment. Because there were no differences in observed results when ethnicity was categorized into five groups instead of two groups, we reclassified ethnicity as European or non-European. Maternal anthropometrics were assessed at time of enrollment. Maternal smoking and alcohol consumption before and during pregnancy were assessed by questionnaires in each trimester and were categorized as none, only until the pregnancy was known, or continued during pregnancy. Month of conception and month of birth were categorized into seasons: winter (December–February), spring (March–May), summer (June–August), and fall (September–November). Road traffic noise exposure was assessed at the home address (in first trimester and at delivery) according to requirements of the European Environmental Noise Directive, and expressed in the noise metric *L*_den_ (day, evening, night), as described in detail elsewhere (van den Hooven et al. 2011). To each participant, we assigned the noise exposure level calculated at the home address at time of the blood sampling (first trimester or delivery).

*Statistical analysis.* Air pollution exposures in each period were categorized into quartiles. The lowest quartile of PM_10_ and NO_2_ exposure was used as the reference group. First, unadjusted and adjusted linear regression models were run to analyze the associations for an interquartile range increase in air pollution exposure in different periods preceding the first-trimester measurement with maternal CRP levels. Maternal CRP concentrations were log-transformed (using the natural log) to obtain a normally distributed outcome variable. We present coefficients from the linear regression analyses for the log-transformed CRP concentrations, multiplied by 100, which can be interpreted in units of percentage differences (Cole 2000). Second, the associations of air pollution exposure quartiles for different periods preceding the first-trimester measurement with elevated maternal CRP levels (> 8 mg/L) were estimated using unadjusted and adjusted logistic regression models. Third, unadjusted and adjusted logistic regression models were run to estimate associations of air pollution exposure quartiles for different periods preceding delivery with elevated fetal CRP levels (> 1 mg/L). Logistic regression models in which air pollution exposure was included as a continuous variable (per 10-μg/m^3^ increase) were considered as test for trend. All models were adjusted for known determinants of CRP levels (maternal age, body mass index, ethnicity, education, parity, smoking, alcohol consumption, and gestational age at measurement) and for road traffic noise exposure (based on home address in first trimester for models on maternal CRP levels or on home address at delivery for models on fetal CRP levels). Models with maternal CRP levels were additionally adjusted for season of conception, and models with fetal CRP levels were additionally adjusted for season of birth. The percentages of missing values within the population for analysis were < 1% for continuous data and < 15% for the categorical data. We applied multiple imputation for missing data in covariates. All measures of association are presented with their 95% confidence intervals (CIs). All statistical analyses were performed using PASW version 17.0 for Windows (PASW Inc.). *p*-Values of < 0.05 and < 0.10 were considered statistically significant and borderline statistically significant, respectively.

## Results

*Subject and exposure characteristics.* The median age of the participants was 30.4 years (Table 1). Most of the women were nulliparous, and 41.2% had completed high education. Median maternal CRP concentration was 4.4 mg/L (range, 0.2–93.8 mg/L); 1,309 women had an elevated CRP concentration (> 8.0 mg/L). Of the neonates, 3,485 (53.5%) had a CRP concentration below the detection limit of 0.2 mg/L; 72 neonates had an elevated CRP concentration (> 1.0 mg/L). Mean maternal exposure levels for the prior week were 30.6 μg/m^3^ for PM_10_ and 40.3 μg/m^3^ for NO_2_ in early pregnancy, and 29.6 μg/m^3^ for PM_10_ and 39.5 μg/m^3^ for NO_2_ at delivery [see Supplemental Material, Table S1 (http://dx.doi.org/10.1289/ehp.1104345)]. Mean air pollution exposure levels for the total pregnancy period were 30.3 μg/m^3^ (range, 23.2–40.9 μg/m^3^) for PM_10_ and 39.9 μg/m^3^ (range, 26.5–56.9 μg/m^3^) for NO_2_. On average, these levels are below the European Union annual limit values (40 μg/m^3^ for both PM_10_ and NO_2_) that are defined for protection of human health (World Health Organization 2006), but a substantial proportion (46%) of the women were exposed to NO_2_ levels higher than this limit value. Correlations among exposure averages for the prior 1, 2, and 4 weeks were moderate to strong (PM_10_, Pearson correlation coefficient *r* = 0.58–0.83; NO_2_, *r* = 0.74–0.89). Correlations between exposure averages for the prior 1, 2, and 4 weeks with exposure averages for the total pregnancy period were lower (PM_10_, *r* = 0.27–0.48; NO_2_, *r* = 0.36–0.51). PM_10_ and NO_2_ levels for the same period were moderately correlated (*r* = 0.35–0.54).

**Table 1:** Subject characteristics (n = 6,508).

Characteristic	Value
Maternal characteristics	
Age at enrollment [years; median (range)]	30.4 (15.4–46.3)
Gestational age at enrollment [weeks; median (range)]	13.1 (5.1–38.4)
Height (cm; mean ± SD)	167.4 ± 7.5
Weight at enrollment [kg; median (range)]	67.0 (37.0–142.0)
Body mass index at enrollment [kg/m2; median (range)]	23.7 (15.2–51.2)
Parity [n (%)]	
Nulliparous	3,592 (55.2)
Multiparous	2,854 (43.9)
Missing	62 (1.0)
Ethnic background [n (%)]	
European	3,624 (55.7)
Non-European	2,483 (38.2)
Missing	401 (6.2)
Highest completed educational level [n (%)]	
No education/primary	626 (9.6)
Secondary	2,701 (41.5)
Higher	2,680 (41.2)
Missing	501 (7.7)
Smoking in pregnancy [n (%)]	
None	4,192 (64.4)
First trimester only	492 (7.6)
Continued	1,002 (15.4)
Missing	822 (12.6)
Alcohol consumption in pregnancy [n (%)]	
None	2,695 (41.4)
First trimester only	779 (12.0)
Continued	2,259 (34.7)
Missing	775 (11.9)
Season of conception [n (%)]	
Winter	1,781 (27.4)
Spring	1,516 (23.3)
Summer	1,521 (23.4)
Fall	1,690 (26.0)
Noise exposure based on home address in first trimester [dB(A); median (range)]	53.1 (45.0–76.0)
Noise exposure based on home address at delivery [dB(A); median (range)]	52.7 (45.0–76.0)
Gestational age at blood sampling [weeks; median (range)]	13.2 (4.5–17.9)
CRP concentration [mg/L; median (range)]	4.4 (0.2–93.8)
CRP concentration > 8.0 mg/L [n (%)]	1,309 (24.8)
Child characteristics	
Gestational age at birth [weeks; median (range)]	40.1 (27.6–43.6)
Birth weight (g; mean ± SD)	3460.7 ± 502.5
CRP concentration > 1.0 mg/L [n (%)]	69 (1.5)
Values are means ± SD, or medians (range) for variables with a skewed distribution, and number of subjects (%) in case of categorical variables.	

**Figure 1:**
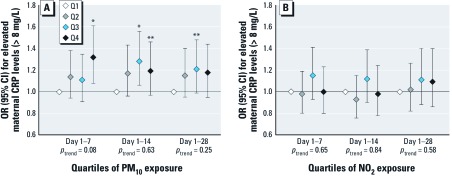
Associations of maternal air pollution exposure with the risks of elevated maternal CRP levels in early pregnancy (*n* = 5,067). Values [ORs (95% CI)] reflect the risk for elevated maternal CRP levels (> 8 mg/L) for each quartile (Q) of PM_10_ exposure (*A*) and NO_2_ exposure (*B*) in different periods preceding the first-trimester measurement compared with the reference group (lowest quartile). Cutoff values for categorization of PM_10_ exposure were < 24.6, 24.6–28.8, 28.8–33.9, and > 33.9 μg/m^3^ for the prior week, < 25.4, 25.4–28.8, 28.8–33.7, and > 33.7 μg/m^3^ for the prior 2 weeks, and < 26.3, 26.3–29.4, 29.4–33.8, and > 33.8 μg/m^3^ for the prior 4 weeks. Cutoff values for NO_2_ exposure were < 33.9, 33.9–39.9, 39.9–46.0, and > 46.0 μg/m^3^ for the prior week, < 35.2, 35.2–40.5, 40.5–45.3, and > 45.3 μg/m^3^ for the prior 2 weeks, and < 35.8, 35.8–40.8, 40.8–44.5, and > 44.5 μg/m^3^ for the prior 4 weeks. Tests for trend were performed by including PM_10_ and NO_2_ exposure as a continuous term (per 10‑μg/m^3^ increase) in the model. Number of subjects classified as having elevated CRP levels is indicated in Supplemental Material, Table S3 (http://dx.doi.org/10.1289/ehp.1104345). Models are adjusted for gestational age at measurement, maternal age, body mass index, parity, ethnicity, education, smoking, alcohol consumption, noise exposure, and season of conception. *p < 0.05; **p < 0.10.

*Air pollution and maternal CRP levels.* We observed nonsignificant, negative percentage changes in maternal CRP levels per interquartile range increase in air pollution exposure preceding the first-trimester measurement in the unadjusted models. Adjustment for covariates attenuated the effect estimates toward the null [see Supplemental Material, Table S2 (http://dx.doi.org/10.1289/ehp.1104345)]. Compared with the lowest quartile, the highest quartile of PM_10_ exposure for the prior week was associated with elevated maternal CRP levels [> 8 mg/L; odds ratio (OR) = 1.32; 95% CI: 1.08, 1.61) (Figure 1A)]. The third and fourth quartiles of PM_10_ exposure for the prior 2 weeks were also associated with elevated CRP (OR = 1.28; 95% CI: 1.06, 1.56; and OR = 1.19; 95% CI: 0.97, 1.46, respectively). However, ORs were comparable for all quartiles, and tests for trend were not significant. Associations of PM_10_ exposure levels for the prior 4 weeks with maternal CRP levels in early pregnancy did not reach statistical significance (Figure 1A). NO_2_ exposure levels for the prior 1, 2, and 4 weeks were not associated with maternal CRP levels in early pregnancy (Figure 1B). When we performed analyses with different cutoff points for CRP (> 10 and > 5 mg/L, *n* = 915 and *n* = 2,316 classified as elevated, respectively), results were comparable (i.e., the same patterns of associations were observed) (data not shown). When we restricted the analyses to 2,403 women with an early CRP measurement (before gestational week 13), we observed similar patterns of associations, although *p*-values were larger for the associations of PM_10_ exposure for the prior 2 and 4 weeks with CRP levels. When we excluded women with preexisting conditions (diagnosed hypertension, diabetes, high cholesterol, chronic heart disorders, or systemic lupus erythematosus; *n* = 179), the results did not change. Results from the sensitivity analyses in nonsmoking women (*n* = 4,192) or in women without illnesses in the first trimester that could indicate a possible infection or inflammation (*n* = 5,792) were similar. Additional adjustment for maternal passive smoking or meteorological conditions on the day of the measurement (24-hr average temperature, maximum temperature, relative humidity, and barometric pressure) did not influence the results either. Associations were comparable when the analyses were restricted to women with a normal body mass index (< 25 kg/m^2^; *n* = 4,103) (data not shown). The unadjusted models showed smaller effect estimates with larger *p*-values (see Supplemental Material, Table S3).

**Figure 2:**
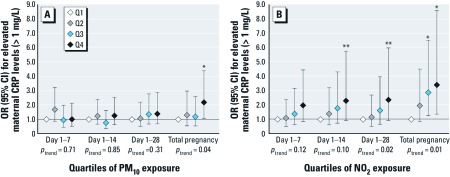
Associations of maternal air pollution exposure with the risks of elevated fetal CRP levels at delivery (*n* = 4,450). Values [ORs (95% CI)] reflect the risk for elevated fetal CRP levels (> 1 mg/L) for each quartile (Q) of PM_10_ exposure (*A*) and NO_2_ exposure (*B*) in different periods preceding delivery compared with the reference group (lowest quartile). Cutoff values for categorization of PM_10_ exposure were < 23.9, 23.9–27.7, 27.7–32.8, and > 32.8 μg/m^3^ for the prior week, < 24.7, 24.7–28.0, 28.0–32.1, and > 32.1 μg/m^3^ for the prior 2 weeks, < 25.6, 25.6–28.5, 28.5–32.8, and > 32.8 μg/m^3^ for the prior 4 weeks, and < 27.8, 27,8–30.0, 30.0–32.9, and > 32.9 μg/m^3^ for total pregnancy. Cutoff values for NO_2_ exposure were < 33.2, 33.2–39.3, 39.3–45.6, and > 45.6 μg/m^3^ for the prior week, < 34.1, 34.1–39.8, 39.8–44.7, > and 44.7 μg/m^3^ for the prior 2 weeks, < 34.7, 34.7–40.2, 40.2–44.1, and > 44.1 μg/m^3^ for the prior 4 weeks, and < 37.2, 37.2–39.6, 39.6–42.3, and > 42.3 μg/m^3^ for total pregnancy. Number of subjects classified as having elevated CRP levels is indicated in Supplemental Material, Table S4 (http://dx.doi.org/10.1289/ehp.1104345). Models are adjusted for gestational age at birth, season of birth, maternal age, body mass index, parity, ethnicity, education, smoking, alcohol consumption, and noise exposure. Tests for trend were performed by including PM_10_ and NO_2_ exposure as a continuous term (per 10-μg/m^3^ increase) in the model. *p < 0.05; **p < 0.10.

*Air pollution and fetal CRP levels.* No consistent associations with fetal CRP levels were observed for maternal PM_10_ exposure for 1, 2, and 4 weeks preceding delivery in the adjusted models (Figure 2A). Compared with the lowest quartile, the fourth quartile of PM_10_ exposure during total pregnancy was associated with elevated fetal CRP levels (> 1 mg/L) at delivery (OR = 2.18; 95% CI: 1.08, 4.38), and a positive trend (*p* = 0.04) was observed as well. Positive, but nonsignificant associations were observed for NO_2_ exposure for the prior 1 and 2 weeks with fetal CRP levels at delivery (Figure 2B), with a monotonic increase in ORs. A positive trend was observed for NO_2_ exposure for the prior 4 weeks and elevated fetal CRP levels (*p* = 0.02). Elevated fetal CRP levels were associated with the third and fourth quartiles of NO_2_ exposure during total pregnancy (OR = 2.85; 95% CI: 1.25, 6.47; and OR = 3.42; 95% CI: 1.36, 8.58, respectively), with a monotonic increase in ORs (*p* = 0.01). When we performed analyses with different cutoff points for fetal CRP (> 0.8 and > 0.4 mg/L, *n* = 85 and *n* = 127 classified as elevated, respectively), results were comparable (data not shown). The same patterns of associations were observed in the sensitivity analyses in nonsmoking women and in mothers without illnesses in first trimester. Additional adjustment for mode of delivery, maternal passive smoking, or meteorological conditions on the day of the measurement did not change the results. When we restricted the analyses to women with a normal body mass index, we observed slightly larger effect estimates for the associations between air pollution and fetal CRP levels (e.g., highest quartiles of total pregnancy exposure: PM_10_, OR = 3.46; 95% CI: 1.18, 10.10; NO_2_, OR = 3.69; 95% CI: 1.04, 12.98). Unadjusted associations for air pollution exposure with elevated fetal CRP were largely similar, although smaller effect estimates with larger *p*-values were observed for total pregnancy exposure [Supplemental Material, Table S4 (http://dx.doi.org/10.1289/ehp.1104345)]. We did not observe consistent associations between the maternal and fetal CRP response to air pollution (data not shown).

## Discussion

In this large population-based prospective cohort study from early pregnancy onward, we observed weak associations for short-term average PM_10_ exposure levels (prior 1 and 2 weeks) with elevated maternal CRP levels in first trimester of pregnancy. Higher long-term average PM_10_ and NO_2_ exposure levels (total pregnancy) were associated with elevated fetal CRP levels at delivery. This study extends previous epidemiological research on air pollution and markers of systemic inflammation in various populations and suggests that maternal air pollution exposure may promote inflammatory processes in the mother and fetus.

*Air pollution and CRP levels during pregnancy.* In normal pregnancy, maternal CRP levels slightly increase as a result of the inflammatory response to the pregnancy (Thornton 2010; von Versen-Hoeynck et al. 2009). This systemic inflammatory response, which is part of the innate immune system, generally peaks during the third trimester (Redman and Sargent 2004). Among pregnant women, a further elevation of CRP levels has been reported in pregnancies complicated by fetal growth restriction, preeclampsia, and preterm delivery (Guven et al. 2009; Lohsoonthorn et al. 2007; Pitiphat et al. 2005; Tjoa et al. 2003). In addition, increased CRP levels in cord blood have been observed in neonates that were born preterm, small for gestational age, or with a low birth weight (Amarilyo et al. 2011; Trevisanuto et al. 2007). Two recent studies in our population showed that elevated maternal CRP levels in first trimester were associated with reductions in third-trimester estimated fetal weight and birth weight and with small size for gestational age at birth (Ernst et al. 2011). In addition, maternal CRP levels were positively associated with diastolic blood pressure, and elevated CRP levels were associated with pregnancy-induced hypertension and preeclampsia, but these associations attenuated toward the null after adjustment for maternal body mass index (de Jonge et al. 2011). These findings indicate a possible link between an enhanced systemic inflammatory response and adverse pregnancy outcomes.

Potential biological pathways through which air pollution, especially PM, may influence pregnancy are induction of oxidative stress and translocation of PM directly in the blood, both resulting in systemic inflammation (Brook et al. 2010). It has been hypothesized that an enhanced systemic inflammatory response may lead to placental inflammation and alterations in maternal immunity (Kannan et al. 2006), or suboptimal placentation (Dejmek et al. 1999), which could predispose to the development of adverse pregnancy outcomes. A number of routinely measured air pollutants [e.g., PM_10_, PM_2.5_ (aerodynamic diamter ≤ 2.5 µm), NO_2_, carbon monoxide (CO), ozone (O_3_), sulfur dioxide (SO_2_)] have been linked to adverse pregnancy outcomes such as preterm birth, low birth weight, and intrauterine growth restriction (Bonzini et al. 2010; Ritz and Wilhelm 2008; Shah et al. 2011), although results differ among studies. In our previous work in the same population, we have shown that maternal exposure to PM_10_ and NO_2_ during pregnancy was associated with measures of fetal growth retardation and a reduced birth weight. Also, elevated PM_10_ exposure levels were associated with increased risks for preterm birth, small size for gestational age at birth (van den Hooven et al. 2012), and pregnancy-induced hypertension (van den Hooven et al. 2011).

In the present study, no statistically significant percent changes in maternal CRP levels in early pregnancy were observed for an interquartile range increase in PM_10_ or NO_2_ exposure. In contrast, weak associations were observed for short-term average PM_10_ exposure with elevated maternal CRP levels (> 8 mg/L). NO_2_ exposure was not associated with elevated maternal CRP levels. Possibly, air pollution–induced increases in maternal CRP levels, if any, might be difficult to detect, because CRP levels already increase in response to the pregnancy.

Thus far, only one previous study has examined associations of maternal air pollution exposure with CRP levels during pregnancy. This study was conducted in 1,696 women in the United States and showed a tendency toward higher risks for elevated CRP levels (> 8 mg/L) for an interquartile range increase in PM_10_ and PM_2.5_ exposure for the prior 22 and 29 days (ORs ranging from 1.18 to 1.32) (Lee et al. 2011). Effect estimates were generally larger in nonsmokers only. Positive but nonsignificant associations were observed for exposure to O_3_, whereas no associations were observed for exposure to NO_2_, CO, and SO_2_. However, the consideration of the spatial variability of air pollutants was limited in this study, because exposure estimates were based on monitoring stations only. Several other studies estimated the impact of air pollution exposure on CRP levels in nonpregnant adults. Positive associations with CRP levels were observed for exposure to PM_10_ (Chuang et al. 2007; Seaton et al. 1999), NO_2_ (Delfino et al. 2009), or markers of primary combustion, including PM_2.5_ (Delfino et al. 2009; Riediker et al. 2004), but other studies reported only weak associations (Diez Roux et al. 2006; Dubowsky et al. 2006) or were not able to detect associations with PM or NO_2_ (Brauner et al. 2008; Forbes et al. 2009; Rudez et al. 2009; Steinvil et al. 2008; Zuurbier et al. 2011).

Considering fetal CRP levels, in the present study elevated fetal CRP levels at delivery were observed in association with higher exposure to PM_10_ and NO_2_ during total pregnancy. No consistent associations were observed for air pollution exposure in shorter exposure periods (1, 2, or 4 weeks), although ORs increased monotonically with higher NO_2_ levels. To our knowledge, this study is the first to examine associations of maternal air pollution exposure with fetal CRP levels. Because CRP does not cross the placenta (Jaye and Waites 1997), elevated CRP levels are considered to reflect hepatic synthesis by the fetus (Raio et al. 2003). The underlying mechanism through which maternal air pollution exposure may lead to an enhanced inflammatory response in the fetus is unclear. It is possible that it might involve systemic and placental inflammation at the maternal side. Alternatively, air pollution might provoke an inflammatory response directly in the fetus, because of either short- or long-term exposure. We did not observe consistent associations between the maternal and fetal CRP response to air pollution (i.e., whether the air pollution effect in the mother was related to the air pollution effect in the fetus). This may be related to the different timing of the measurements (early pregnancy vs. delivery). We could not examine the possibility that acute maternal infections contributed to elevated fetal CRP levels, because information on third-trimester maternal infections was not available. Future studies are needed to confirm our findings and to explore the underlying mechanisms.

CRP increases rapidly after an inflammatory trigger. Most previous studies on air pollution and CRP levels estimated associations with relatively acute exposures (same-day or multiday averages). Information on the impact of longer averages of air pollution is limited. Possibly, exposure to high air pollution concentrations during a few weeks or months may cause chronically elevated CRP levels in mother and fetus. Effect estimates for associations between air pollution and elevated fetal CRP levels were slightly stronger in the subgroup of women with a normal body mass index. It is known that body mass index is an important determinant of CRP levels in pregnant women, and previous studies have reported increased levels of inflammatory markers (including CRP) in overweight and obese women (Madan et al. 2009; Visser et al. 1999). The increased inflammatory response in overweight and obese women possibly masks the effects of air pollution on maternal and fetal CRP levels.

This study was performed in an urban area that is characterized by high emissions from road traffic, shipping, households, and industry. No information was available on pollutants other than PM_10_ and NO_2_. Mean exposure levels in previous studies that examined associations between air pollution and CRP levels varied substantially. Compared with our study, reported PM_10_ levels were lower in studies in the United Kingdom and the United States (Forbes et al. 2009; Lee et al. 2011; Seaton et al. 1999), similar in another study in Rotterdam, the Netherlands (Rudez et al. 2009), and higher in studies in Taiwan and Israel (Chuang et al. 2007; Steinvil et al. 2008). Reported NO_2_ exposure levels were (slightly) lower in previous studies in Taiwan, Israel, the United Kingdom, and the United States (Chuang et al. 2007; Dubowsky et al. 2006; Forbes et al. 2009; Lee et al. 2011; Steinvil et al. 2008), similar in another Dutch study (Rudez et al. 2009), and higher in a study in Los Angeles, United States (Delfino et al. 2009). However, these comparisons should be considered with caution because of the different averaging periods. Furthermore, adverse health effects associated with PM_10_ and NO_2_ exposure are not necessarily caused by these pollutants but may be caused by other compounds present in the complex air pollution mixture that may differ among geographic locations.

*Methodological considerations.* An important strength of this study is the population-based cohort, which included a large number of participants studied from early pregnancy onward. Furthermore, we collected detailed information on many potential confounding factors, such as maternal educational level, ethnicity, body mass index, smoking, alcohol consumption, and noise exposure. However, residual confounding due to unmeasured variables might still be an issue.

Many previous studies have not addressed both intraurban and temporal contrasts in air pollutants. A few earlier studies on CRP levels in nonpregnant adults considered spatiotemporal variation, either by controlling exposure in an exposure chamber or by measuring personal, indoor-home, or outdoor-home concentrations (Brauner et al. 2008; Delfino et al. 2008; Dubowsky et al. 2006; Riediker et al. 2004; Seaton et al. 1999; Zuurbier et al. 2011). However, these studies were based on relatively small sample sizes (*n* < 150) and were often conducted in elderly or diseased subjects (Delfino et al. 2008; Dubowsky et al. 2006; Seaton et al. 1999). In our study, we were able to consider detailed spatial and temporal variation in exposure by using a combination of dispersion modeling and continuous monitoring. Moreover, we were able to account for residential mobility of the women during pregnancy.

We should still acknowledge the possibility of misclassification of air pollution exposure, because exposures were only estimated at the home address, and study participants did not spend all of their time at home. No information was available on other locations or other types of exposure (e.g., occupational, commuting, or indoor sources). This limitation should be taken into account when interpreting the results. Ideally, information on time–activity patterns should be considered when examining the associations for air pollution with health outcomes (Nethery et al. 2009; Ritz and Wilhelm 2008), but unfortunately this information was not available. Whether and in which direction this possible misclassification has affected our effect estimates is not clear. Nevertheless, because pregnant women are likely to spend more time at home than are nonpregnant individuals, especially in the last stage of pregnancy (Nethery et al. 2009), the extent of the possible misclassification may be less than in nonpregnant adults.

The present study was based on single blood measurements. Future studies that longitudinally follow the changes of CRP levels during pregnancy in relation to air pollution exposure are recommended.

## Conclusions

In a population-based prospective cohort study in the Netherlands, we showed that short-term maternal PM_10_ exposure was modestly associated with elevated maternal CRP levels in early pregnancy and that long-term maternal PM_10_ and NO_2_ exposure during pregnancy was associated with elevated fetal CRP levels at delivery. Our results suggest that air pollution exposure may lead to maternal and fetal inflammatory responses. More research is needed to confirm these findings, to examine the underlying mechanisms, and to explore the consequences.

## Supplemental Material

(119 KB) PDF

## References

[r1] Amarilyo G, Oren A, Mimouni FB, Ochshorn Y, Deutsch V, Mandel D (2011). Increased cord serum inflammatory markers in small-for-gestational-age neonates.. J Perinatol.

[r2] Bonzini M, Carugno M, Grillo P, Mensi C, Bertazzi PA, Pesatori AC (2010). Impact of ambient air pollution on birth outcomes: systematic review of the current evidences.. Med Lav.

[r3] Brauner EV Moller P Barregard L Dragsted LO Glasius M Wahlin P 2008 Exposure to ambient concentrations of particulate air pollution does not influence vascular function or inflammatory pathways in young healthy individuals. Part Fibre Toxicol 5 13; 10.1186/1743-8977-5-13 [Online 6 October 2008] 18837984 PMC2579917

[r4] Brook RD, Rajagopalan S, Pope CA, Brook JR, Bhatnagar A, Diez-Roux AV (2010). Particulate matter air pollution and cardiovascular disease: an update to the scientific statement from the American Heart Association.. Circulation.

[r5] Catov JM, Bodnar LM, Ness RB, Barron SJ, Roberts JM (2007). Inflammation and dyslipidemia related to risk of spontaneous preterm birth.. Am J Epidemiol.

[r6] Chuang KJ, Chan CC, Su TC, Lee CT, Tang CS (2007). The effect of urban air pollution on inflammation, oxidative stress, coagulation, and autonomic dysfunction in young adults.. Am J Respir Crit Care Med.

[r7] Cole TJ (2000). Sympercents: symmetric percentage differences on the 100 log(e) scale simplify the presentation of log transformed data.. Stat Med.

[r8] Dejmek J, Selevan SG, Benes I, Solanský I, Šrám RJ (1999). Fetal growth and maternal exposure to particulate matter during pregnancy.. Environ Health Perspect.

[r9] de Jonge LL, Steegers EA, Ernst GD, Lindemans J, Russcher H, Hofman A (2011). C-reactive protein levels, blood pressure and the risks of gestational hypertensive complications: the Generation R Study.. J Hypertens.

[r10] Delfino RJ, Staimer N, Tjoa T, Gillen DL, Polidori A, Arhami M (2009). Air pollution exposures and circulating biomarkers of effect in a susceptible population: clues to potential causal component mixtures and mechanisms.. Environ Health Perspect.

[r11] Delfino RJ, Staimer N, Tjoa T, Polidori A, Arhami M, Gillen DL (2008). Circulating biomarkers of inflammation, antioxidant activity, and platelet activation are associated with primary combustion aerosols in subjects with coronary artery disease.. Environ Health Perspect.

[r12] Diez Roux AV, Auchincloss AH, Astor B, Barr RG, Cushman M, Dvonch T (2006). Recent exposure to particulate matter and C-reactive protein concentration in the multi-ethnic study of atherosclerosis.. Am J Epidemiol.

[r13] Dubowsky SD, Suh H, Schwartz J, Coull BA, Gold DR (2006). Diabetes, obesity, and hypertension may enhance associations between air pollution and markers of systemic inflammation.. Environ Health Perspect.

[r14] Ernst GD, de Jonge LL, Hofman A, Lindemans J, Russcher H, Steegers EA (2011). C-reactive protein levels in early pregnancy, fetal growth patterns, and the risk for neonatal complications: the Generation R Study.. Am J Obstet Gynecol.

[r15] Forbes LJ, Patel MD, Rudnicka AR, Cook DG, Bush T, Stedman JR (2009). Chronic exposure to outdoor air pollution and markers of systemic inflammation.. Epidemiology.

[r16] Gabay C, Kushner I. (1999). Acute-phase proteins and other systemic responses to inflammation.. N Engl J Med.

[r17] Guven MA, Coskun A, Ertas IE, Aral M, Zencirci B, Oksuz H (2009). Association of maternal serum CRP, IL-6, TNF-alpha, homocysteine, folic acid and vitamin B12 levels with the severity of preeclampsia and fetal birth weight.. Hypertens Pregnancy.

[r18] Jaddoe VW, Bakker R, van Duijn CM, van der Heijden AJ, Lindemans J, Mackenbach JP (2007). The Generation R Study Biobank: a resource for epidemiological studies in children and their parents.. Eur J Epidemiol.

[r19] Jaddoe VW, van Duijn CM, van der Heijden AJ, Mackenbach JP, Moll HA, Steegers EA (2010). The Generation R Study: design and cohort update 2010.. Eur J Epidemiol.

[r20] Jaye DL, Waites KB (1997). Clinical applications of C-reactive protein in pediatrics.. Pediatr Infect Dis J.

[r21] Kannan S, Misra DP, Dvonch JT, Krishnakumar A (2006). Exposures to airborne particulate matter and adverse perinatal outcomes: a biologically plausible mechanistic framework for exploring potential effect modification by nutrition.. Environ Health Perspect.

[r22] Kordek A, Halasa M, Podraza W. (2008). Early detection of an early onset infection in the neonate based on measurements of procalcitonin and C-reactive protein concentrations in cord blood.. Clin Chem Lab Med.

[r23] Lee PC, Talbott EO, Roberts JM, Catov JM, Sharma RK, Ritz B (2011). Particulate air pollution exposure and C-reactive protein during early pregnancy.. Epidemiology.

[r24] Lohsoonthorn V, Qiu C, Williams MA (2007). Maternal serum C-reactive protein concentrations in early pregnancy and subsequent risk of preterm delivery.. Clin Biochem.

[r25] Madan JC, Davis JM, Craig WY, Collins M, Allan W, Quinn R (2009). Maternal obesity and markers of inflammation in pregnancy.. Cytokine.

[r26] Netherlands Ministry of Infrastructure and the Environment (2007). Regeling beoordeling Luchtkwaliteit 2007 (Air Quality Decree 2007) [in Dutch].. http://wetten.overheid.nl/BWBR0022817.

[r27] Nethery E, Brauer M, Janssen P. (2009). Time-activity patterns of pregnant women and changes during the course of pregnancy.. J Expo Sci Environ Epidemiol.

[r28] Pitiphat W, Gillman MW, Joshipura KJ, Williams PL, Douglass CW, Rich-Edwards JW (2005). Plasma C-reactive protein in early pregnancy and preterm delivery.. Am J Epidemiol.

[r29] Raio L, Ghezzi F, Mueller MD, McDougall J, Malek A (2003). Evidence of fetal C-reactive protein urinary excretion in early gestation.. Obstet Gynecol.

[r30] Redman CW, Sargent IL (2004). Preeclampsia and the systemic inflammatory response.. Semin Nephrol.

[r31] Riediker M, Cascio WE, Griggs TR, Herbst MC, Bromberg PA, Neas L (2004). Particulate matter exposure in cars is associated with cardiovascular effects in healthy young men.. Am J Respir Crit Care Med.

[r32] Ritz B, Wilhelm M. (2008). Ambient air pollution and adverse birth outcomes: methodologic issues in an emerging field.. Basic Clin Pharmacol Toxicol.

[r33] Roberts WL, Moulton L, Law TC, Farrow G, Cooper-Anderson M, Savory J (2001). Evaluation of nine automated high-sensitivity C-reactive protein methods: implications for clinical and epidemiological applications. Part 2.. Clin Chem.

[r34] Rudez G, Janssen NA, Kilinc E, Leebeek FW, Gerlofs-Nijland ME, Spronk HM (2009). Effects of ambient air pollution on hemostasis and inflammation.. Environ Health Perspect.

[r35] Seaton A, Soutar A, Crawford V, Elton R, McNerlan S, Cherrie J (1999). Particulate air pollution and the blood.. Thorax.

[r36] Shah PS, Balkhair T, Knowledge Synthesis Group on Determinants of Preterm/LBW Births (2011). Air pollution and birth outcomes: a systematic review.. Environ Int.

[r37] Slama R, Darrow L, Parker J, Woodruff TJ, Strickland M, Nieuwenhuijsen M (2008). Meeting report: atmospheric pollution and human reproduction.. Environ Health Perspect.

[r38] Steinvil A, Kordova-Biezuner L, Shapira I, Berliner S, Rogowski O. (2008). Short-term exposure to air pollution and inflammation-sensitive biomarkers.. Environ Res.

[r39] Thornton CA (2010). Immunology of pregnancy.. Proc Nutr Soc.

[r40] Tjoa ML, van Vugt JM, Go AT, Blankenstein MA, Oudejans CB, van Wijk IJ (2003). Elevated C-reactive protein levels during first trimester of pregnancy are indicative of preeclampsia and intrauterine growth restriction.. J Reprod Immunol.

[r41] Trevisanuto D, Doglioni N, Altinier S, Zaninotto M, Plebani M, Zanardo V. (2007). High-sensitivity C-reactive protein in umbilical cord of small-for-gestational-age neonates.. Neonatology.

[r42] van den Hooven EH, de Kluizenaar Y, Pierik FH, Hofman A, van Ratingen SW, Zandveld PY (2011). Air pollution, blood pressure, and the risk of hypertensive complications during pregnancy: the Generation R Study.. Hypertension.

[r43] van den Hooven EH, Pierik FH, de Kluizenaar Y, Willemsen SP, Hofman A, van Ratingen SW (2012). Air pollution exposure during pregnancy, ultrasound measures of fetal growth, and adverse birth outcomes: a prospective cohort study.. Environ Health Perspect.

[r44] Visser M, Bouter LM, McQuillan GM, Wener MH, Harris TB (1999). Elevated C-reactive protein levels in overweight and obese adults.. JAMA.

[r45] von Versen-Hoeynck FM, Hubel CA, Gallaher MJ, Gammill HS, Powers RW (2009). Plasma levels of inflammatory markers neopterin, sialic acid, and C-reactive protein in pregnancy and preeclampsia.. Am J Hypertens.

[r46] World Health Organization (2006). WHO Air Quality Guidelines for Particulate Matter, Ozone, Nitrogen Dioxide and Sulfur Dioxide. Global Update 2005. Summary of Risk Assessment.. http://whqlibdoc.who.int/hq/2006/WHO_SDE_PHE_OEH_06.02_eng.pdf.

[r47] Zuurbier M, Hoek G, Oldenwening M, Meliefste K, Krop E, van den Hazel P (2011). In-traffic air pollution exposure and CC16, blood coagulation, and inflammation markers in healthy adults.. Environ Health Perspect.

