# Supplementary material for: Chronic Air Pollution Exposure during Pregnancy and Maternal and Fetal C-Reactive Protein Levels: The Generation R Study
Source: Environ Health Perspect. 2012 Feb 3;120(5):746–51. doi: 10.1289/ehp.1104345 (PMC3346784; doi:10.1289/ehp.1104345)
Supplement: (119 KB) PDF [file ehp.1104345.s001.508.pdf]

Supplemental Material

Chronic Air Pollution Exposure during Pregnancy and Maternal and Fetal C-reactive Protein Levels. The Generation R Study

Edith H. van den Hooven, Yvonne de Kluizenaar, Frank H Pierik, Albert Hofman,

Sjoerd W. van Ratingen, Peter Y.J. Zandveld, Jan Lindemans, Henk Russcher,

Eric A.P. Steegers, Henk M.E. Miedema, Vincent W.V. Jaddoe

Table of Contents

2 Figure S1. Population for analysis

3 Table S1. Distribution of PM_{10} and NO_2 exposure levels for different periods

4 Table S2. Unadjusted and adjusted percentage changes in C-reactive protein levels in early pregnancy for an interquartile range increase in maternal air pollution exposure

5 Table S3. Unadjusted associations of maternal air pollution exposure with the risk of elevated maternal C-reactive protein levels in early pregnancy

6 Table S4. Unadjusted associations of maternal air pollution exposure with the risk of elevated fetal C-reactive protein levels at delivery

Supplemental Material, Figure S1. Population for analysis.

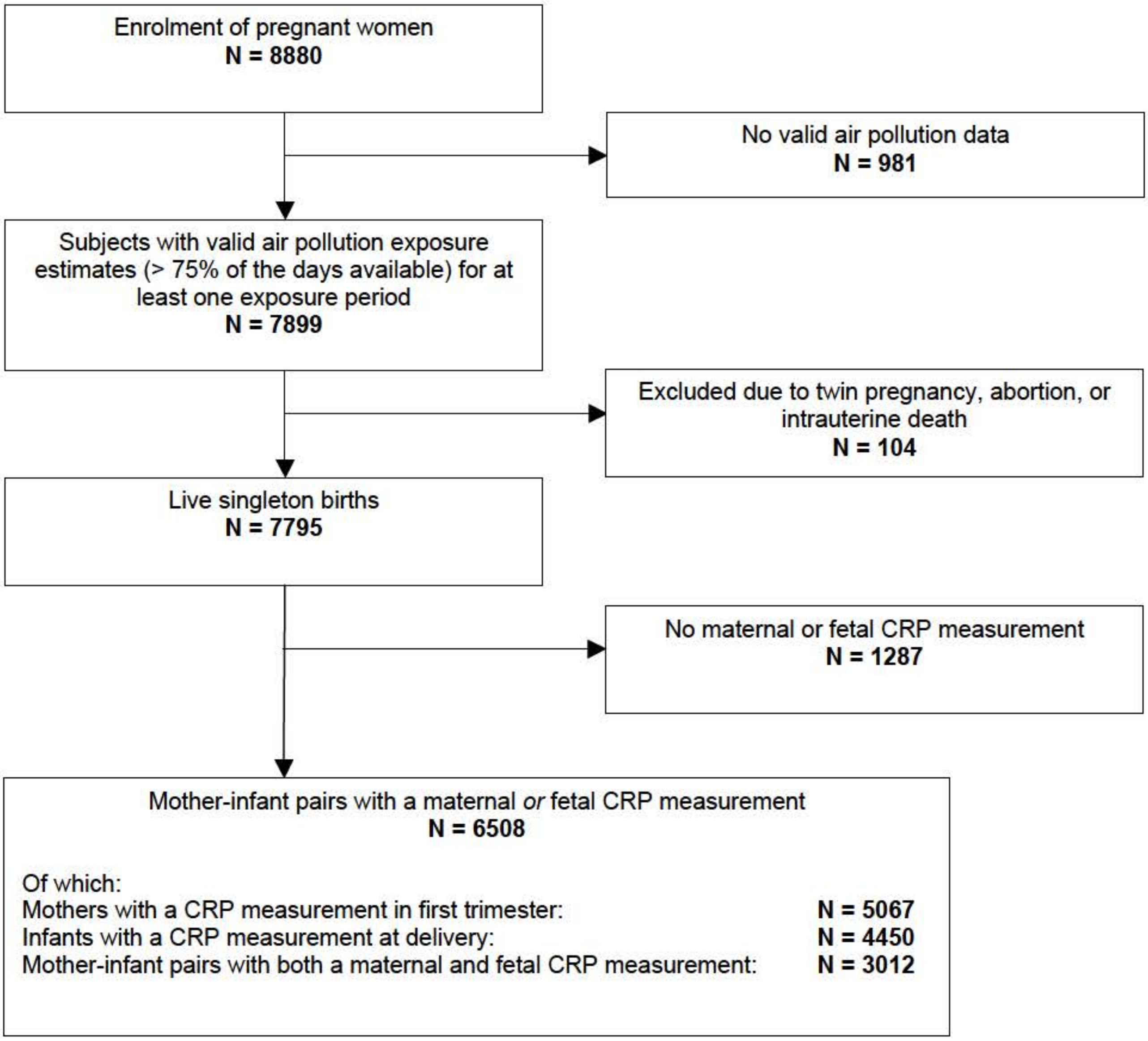

Supplemental Material, Table S1. Distribution of PM_{10} and NO_2 exposure levels for different periods [a].

	Minimum	25th percentile	Mean	Median	75th percentile	Maximum
**Early pregnancy**						
**PM_{10} exposure** (µg/m^3)						
Prior day 1-7	16.3	24.6	30.6	28.8	33.9	66.2
Prior day 1-14	18.8	25.4	30.6	28.8	33.7	58.0
Prior day 1-28	20.2	26.3	30.6	29.4	33.8	49.5
**NO_2 exposure** (µg/m^3)						
Prior day 1-7	16.6	33.9	40.3	39.9	46.0	73.5
Prior day 1-14	16.9	35.2	40.4	40.5	45.3	67.4
Prior day 1-28	19.8	35.8	40.4	40.8	44.5	65.5
**Delivery**						
**PM_{10} exposure** (µg/m3)						
Prior day 1-7	15.2	23.9	29.6	27.7	32.8	62.5
Prior day 1-14	16.9	24.7	29.5	28.0	32.1	53.6
Prior day 1-28	20.1	25.6	29.6	28.5	32.8	44.5
Total pregnancy	23.2	27.8	30.3	30.0	32.9	40.9
**NO_2 exposure** (µg/m3)						
Prior day 1-7	13.0	33.2	39.5	39.3	45.6	69.1
Prior day 1-14	15.3	34.1	39.5	39.8	44.7	67.2
Prior day 1-28	17.6	34.7	39.5	40.2	44.1	62.8
Total pregnancy	26.5	37.2	39.9	39.6	42.3	56.9

[a] Air pollution exposure was estimated for different periods preceding blood sampling: one week (day 1-7), two weeks (day 1-14), and four weeks (day 1-28). Additionally, exposure was estimated for the total pregnancy period (conception until delivery).

Supplemental Material, Table S2. Unadjusted and adjusted percentage changes in maternal C-reactive protein levels in early pregnancy for an interquartile range increase in maternal air pollution exposure (N=5067).

	IQR (μg/m³)	N [a]	Maternal CRP levels Unadjusted percentage change [b] (95% range)	Maternal CRP levels Adjusted percentage change [c] (95% range)
**PM_{10}**				
**Day 1-7**	9.23	5057	0.0 (-2.7, 2.6)	0.9 (-1.6, 3.4)
**Day 1-14**	8.32	5057	-2.1 (-5.0, 0.6)	-1.1 (-3.8, 1.6)
**Day 1-28**	7.45	5037	-1.8 (-5.1, 1.5)	0.3 (-2.9, 3.6)
**NO_2**				
**Day 1-7**	12.11	5065	-0.9 (-4.4, 2.5)	0.4 (-3.1, 3.9)
**Day 1-14**	10.07	5057	-1.3 (-4.6, 2.1)	-0,4 (-4.1, 3.3)
**Day 1-28**	8.69	5047	-1.2 (-4.5, 2.2)	-0.4 (-4.4, 3.6)

Values are log-transformed regression coefficients and reflect the percent change (95% range) in maternal CRP levels in early pregnancy per interquartile range increase in air pollution exposure in different periods preceding the first trimester measurement.

[a] Differences in the number of subjects are due to missing air pollution data for the specific periods.

[b] Models are adjusted for gestational age at measurement.

[c] Models are adjusted for gestational age at measurement, maternal age, body mass index, parity, ethnicity, education, smoking, alcohol consumption, noise exposure, and season of conception.

Supplemental Material, Table S3. Unadjusted associations of maternal air pollution exposure with the risk of elevated maternal C-reactive protein levels in early pregnancy (N=5067).

		**Risk of elevated maternal CRP levels (>8 mg/L) per PM_{10} quartile** Odds ratio (95% CI) *(n of cases)*	**Risk of elevated maternal CRP levels (>8 mg/L) per NO_2 quartile** Odds ratio (95% CI) *(n of cases)*
**Day 1-7**		N = 5057	N = 5065
	1st quartile	*Reference* *(n = 290)*	*Reference* *(n = 323)*
	2nd quartile	1.14 (0.95, 1.37) *(n = 320)*	0.92 (0.77, 1.11) *(n =303)*
	3rd quartile	1.06 (0.88, 1.28) *(n = 304)*	1.01 (0.84, 1.21) *(n = 324)*
	4th quartile	1.25 (1.04, 1.50) * *(n = 344)*	0.94 (0.78, 1.13) *(n = 307)*
	Trend test [a]	*1.04 (0.97, 1.12)*	*0.98 (0.91, 1.05)*
	P for trend	*0.23*	*0.54*
**Day 1-14**		N = 5057	N = 5057
	1st quartile	*Reference* *(n = 294)*	*Reference* *(n = 323)*
	2nd quartile	1.12 (0.93, 1.34) *(n = 318)*	0.89 (0.74, 1.07) *(n = 296)*
	3rd quartile	1.18 (0.98, 1.41) ‡ *(n = 331)*	1.04 (0.87, 1.25) *(n = 334)*
	4th quartile	1.08 (0.90, 1.30) *(n = 311)*	0.91 (0.76, 1.09) *(n = 299)*
	Trend test [a]	*1.10 (1.07, 1.14)*	*0.96 (0.89, 1.05)*
	P for trend	*0.87*	*0.39*
**Day 1-28**		N = 5037	N = 5047
	1st quartile	*Reference* *(n = 295)*	*Reference* *(n = 315)*
	2nd quartile	1.11 (0.93, 1.34) *(n = 321)*	0.96 (0.80, 1.15) *(n = 306)*

3rd quartile	1.12 (0.94, 1.35)<br>*(n = 321)*	1.01 (0.84, 1.21)<br>*(n = 319)*
4th quartile	1.05 (0.88, 1.27)<br>*(n = 309)*	0.97 (0.81, 1.17)<br>*(n = 308)*
Trend test [a]	*1.01 (0.90, 1.12)*	*0.97 (0.88, 1.07)*
P for trend	*0.93*	*0.54*

* $p < 0.05$

‡ $p < 0.10$

Values are odds ratios (95% CI) and reflect the risk for elevated maternal C-reactive protein levels (>8 mg/L) for each quartile of air pollution exposure in different periods preceding the first trimester measurement as compared to the reference group (lowest quartile). Cut-off values for categorization of PM_{10} exposure were <24.6, 24.6-28.8, 28.8-33.9, >33.9 μg/m^3 for the prior week, <25.4, 25.4-28.8, 28.8-33.7, >33.7 μg/m^3 for the prior two weeks, and <26.3, 26.3-29.4, 29.4-33.8, >33.8 μg/m^3 for the prior four weeks. Cut-off values for NO_2 exposure were <33.9, 33.9-39.9, 39.9-46.0, >46.0 μg/m^3 for the prior week, <35.2, 35.2-40.5, 40.5-45.3, >45.3 μg/m^3 for the prior two weeks, and <35.8, 35.8-40.8, 40.8-44.5, >44.5 μg/m^3 for the prior four weeks. Differences in the number of subjects are due to missing air pollution data for the specific periods. Models are adjusted for gestational age at measurement.

[a] Tests for trend were performed by including PM_{10} and NO_2 exposure as a continuous term (per 10 μg/m^3 increase) in the model.

Supplemental Material, Table S4. Unadjusted associations of maternal air pollution exposure with the risk of elevated fetal C-reactive protein levels at delivery (N=4450).

		**Risk of elevated fetal CRP levels (>1 mg/L) per PM_{10} quartile** Odds ratio (95% CI) *(n of cases)*	**Risk of elevated fetal CRP levels (>1 mg/L) per NO_2 quartile** Odds ratio (95% CI) *(n of cases)*
**Day 1-7**		N = 4422	N = 4420
	1st quartile	*Reference* *(n = 15)*	*Reference* *(n =13)*
	2nd quartile	1.62 (0.85, 3.10) *(n = 25)*	1.20 (0.57, 2.54) *(n = 15)*
	3rd quartile	0.90 (0.43, 1.87) *(n = 14)*	1.45 (0.70, 3.01) *(n = 17)*
	4th quartile	0.96 (0.46, 2.00) *(n = 14)*	1.87 (0.94, 3.72) ‡ *(n = 23)*
	Trend test [a]	*0.92 (0.68, 1.24)*	*1.24 (0.95, 1.62)*
	P for trend	*0.58*	*0.11*
**Day 1-14**		N = 4410	N = 4421
	1st quartile	*Reference* *(n = 17)*	*Reference* *(n = 12)*
	2nd quartile	1.19 (0.62, 2.29) *(n = 20)*	1.41 (0.66, 2.99) *(n = 16)*
	3rd quartile	0.73 (0.35, 1.54) *(n = 12)*	1.71 (0.82, 3.53) *(n = 19)*
	4th quartile	1.14 (0.59, 2.20) *(n = 19)*	1.88 (0.92, 3.84) ‡ *(n = 21)*
	Trend test [a]	*0.98 (0.70, 1.39)*	*1.26 (0.93, 1.70)*
	P for trend	*0.91*	*0.13*
**Day 1-28**		N = 4398	N = 4413
	1st quartile	*Reference* *(n = 15)*	*Reference* *(n =14)*
	2nd quartile	1.06 (0.52, 2.16) *(n = 16)*	1.04 (0.50, 2.20) *(n = 14)*

	3rd quartile	1.30 (0.66, 2.58) *(n = 19)*	1.38 (0.68, 2.78) *(n = 18)*
	4th quartile	1.19 (0.60, 2.37) *(n = 18)*	1.66 (0.85, 3.27) *(n = 22)*
	Trend test [a]	*1.09 (0.71, 1.69)*	*1.32 (0.94, 1.87)*
	P for trend	*0.69*	*0.11*
**Total pregnancy**		N = 4123	N = 4121
	1st quartile	*Reference* *(n = 13)*	*Reference* *(n = 9)*
	2nd quartile	0.90 (0.40, 2.01) *(n = 11)*	1.70 (0.75, 3.87) *(n = 16)*
	3rd quartile	1.03 (0.48, 2.20) *(n = 14)*	2.10 (0.95, 4.63) ‡ *(n = 20)*
	4th quartile	1.85 (0.94, 3.63) ‡ *(n = 25)*	1.85 (0.83, 4.13) *(n = 18)*
	Trend test [a]	*2.07 (0.95, 4.55)*	*1.42 (0.79, 2.54)*
	P for trend	0.07	*0.25*

* p < 0.05

‡ p < 0.10

Values are odds ratios (95% CI) and reflect the risk for elevated fetal C-reactive protein levels (>1 mg/L) for each quartile of air pollution exposure in different periods preceding delivery as compared to the reference group (lowest quartile). Cut-off values for categorization of PM_{10} exposure were <23.9, 23.9-27.7, 27.7-32.8, >32.8 μg/m^3 for the prior week, <24.7, 24.7-28.0, 28.0-32.1, >32.1 μg/m^3 for the prior two weeks, <25.6, 25.6-28.5, 28.5-32.8, >32.8 μg/m^3 for the prior four weeks, and <27.8, 27,8-30.0, 30.0-32.9, >32.9 μg/m^3 for total pregnancy. Cut-off values for NO_2 exposure were <33.2, 33.2-39.3, 39.3-45.6, >45.6 μg/m^3 for the prior week, <34.1, 34.1-39.8, 39.8-44.7, >44.7 μg/m^3 for the prior two weeks, <34.7, 34.7-40.2, 40.2-44.1, >44.1 μg/m^3 for the prior four weeks, and <37.2, 37.2-39.6, 39.6-42.3, >42.3 μg/m^3 for total pregnancy. Differences in the number of subjects are due to missing air pollution data for the specific periods. Models are adjusted for gestational age at birth.

[a] Tests for trend were performed by including PM_{10} and NO_2 exposure as a continuous term (per 10 μg/m^3 increase) in the model.
